# Potential utility of a non-invasive menstrual blood immunophenotype analysis in reproductive medicine

**DOI:** 10.1530/RAF-22-0047

**Published:** 2022-09-16

**Authors:** Kevin Marron, Conor Harrity

**Affiliations:** 1 Sims IVF Clinic, Clonskeagh, Dublin, Ireland; 2 RCSI University of Medicine and Health Sciences, Dublin, Ireland; 3 Beaumont Hospital, Dublin, Ireland

**Keywords:** menstrual blood, immunophenotype, ART, lymphocytes

## Abstract

The purpose of this study was to determine if a comprehensive flow cytometry panel could be used to assess immunophenotype profiles in menstrual blood of patients experiencing reproductive failure and age-matched controls of proven fertility. Menstrual blood samples of 58 recurrent pregnancy loss and repeated implantation failure patients, along with 15 age-matched controls of proven fertility, were obtained within the first 24 h of the onset of menstruation to non-invasively assess the local immunophenotype. Using a comprehensive multi-parameter flow panel, the lymphocyte sub-populations were described and compared. In relation to well-established peripheral blood immunophenotyping values, distinct lymphocyte population differences were noted between the subgroups. The ratios of CD4+ and CD8+ T-cells were inverted in relation to peripheral blood, and uterine natural killler (NK) cells represented by CD56^bright^ were distinctly visualised, emphasising the distinction between menstrual and peripheral blood. In relation to controls, there were marked increases in CD3^+ve^ T-cells (*P* = 0.009), CD4:CD8 ratio (*P* = 0.004), CD19 B-cells (*P* = 0.026) and CD56^dim^ NK cells (*P* = 0.002) in the reproductive failure cases. The study shows that flow cytometric evaluation could provide a rapid and objective analysis of lymphocyte subpopulations in many forms of tissue and fluid. The findings show significant variations in cellular composition of immune cells indicating a distinct compartment, with differences between cases and controls. Immunological assessment of the menstrual blood immunophenotype, in clinically appropriate patients, may provide insight into the aetiology of adverse reproductive outcome, without the risks and inconveniences associated with a more invasive endometrial biopsy.

**Lay summary:**

Unexplained infertility is a difficult issue for patients and physicians, but despite diagnostic strides and innovative methods, there are no clear solutions. The involvement of an overactive or underactive immune system in selected cases is undeniable, and the endometrial lining is the most relevant area for investigation because this is where the embryo implants. Endometrial investigations, however, are highly invasive, involve medication and have to be done at the right time. The method described and evaluated here is an alternative assessment which avoids these difficulties and can be used in a clinical setting.

## Introduction

Research into local endometrial immunological variations as causative factors for reproductive failure is widely considered to be controversial (Thum *et al.* 2005). Natural killer (NK) cell assessment has been widely used by some physicians over the last 20 years despite a lack of conclusive evidence, and many treatments dispensed based on these results (King *et al.* 2010, Sacks 2015, Marron *et al.* 2018*a*). There is much debate regarding the most relevant compartment to evaluate, blood or the luteal phase endometrium. Endometrial tissue has been subject to varied histologic and immunohistochemical evaluations, and as a result, there is growing knowledge about the developmental changes that occur and the resident immune populations (Loke *et al.* 1995, Lachapelle *et al.* 1996, Hanna *et al.* 2006, Lédée *et al.* 2016). The peripheral blood and endometrial mucosal compartments represent diverse immune populations with different functions (Sathaliyawala *et al.* 2013, Rebuli *et al.* 2018, Pattacini *et al.* 2019, Woodward Davis *et al.* 2021). NK cells of the uterine subtype (CD16-, CD56^bright^) have a primary function to facilitate implantation and pregnancy, while the peripheral blood subtype (CD16+ CD56^dim^) is noted for their cytotoxic nature and ability to directly interact with non-self-target cells to devastating effect (Seshadri & Sunkara 2014).

With peripheral blood evaluations, percentage cell distribution values are thought to be easily influenced by external dynamics, so are frequently open to criticism within reproductive medicine (Maecker *et al.* 2012, Moffett & Shreeve 2016, Marron *et al.* 2018*b*). These issues appear to have less impact on luteal phase endometrial tissue, but questions persist regarding the specific functions of the various players, as immunophenotypes across these various compartments appear diverse. Direct examination of the endometrial composition in a non-conceptual cycle by biopsy has the potential to be more relevant than a blood evaluation, as it should better represent the materno–fetal interface where trophoblastic invasion and allo-immune recognition occurs (Marron *et al.* 2018*b*, Yang *et al.* 2019). However, the invasive nature of the test, the need for antibiotic cover, risk of miscarriage in a spontaneous pregnancy and delay of the subsequent treatment cycle open possibilities and benefits for alternative techniques.

Menstrual blood evaluation provides a potential non-invasive approach to assess the endometrial immunological environment (van der Molen *et al.* 2013). This technique can analyse the constituent populations without needing a mid-luteal biopsy during the implantation window and the associated risks. This method does have some inherent difficulties in the timing of collection, contamination with mononuclear or polymorphonuclear cells, as well as an increased peripheral blood contribution as menstruation continues. This latter issue could potentially render this technique less useful for the determination of tissue-resident immune cells. However, if performed within a tightly controlled window of 24 h post menstruation proper, the effects of this contamination can be limited, improving the standardisation and clinical utility of this technique.

Elevated endometrial levels of pNK, NK-T and uNK, or modified CD8 expression, have been associated with patients suffering from recurrent pregnancy loss (Southcombe *et al.* 2017, Marron *et al.* 2018*b*) and low levels of CD8 are implicated in repeat implantation failure (El-Shahawy 2016, Marron *et al.* 2018*b*). Menstrual blood evaluation on the other hand has been relatively under-utilised and represents, under strict collection protocols, a readily available source of material that mimics maternal endometrial immune status. This technique may potentially be expanded to evaluate a wide spectrum, such as in proteomics, microbiome or endometritis assessment, direct cytokine evaluations and other potential but as yet unproven markers.

## Methodology

Our laboratory has an extensive history of performing reproductive immunophenotypes on peripheral blood and endometrial biopsy subsets, developing reference ranges for these using previously described techniques (Marron *et al.* 2018*b*, Marron & Harrity 2019). Recurrent pregnancy loss (RPL) was classified as two or more consecutive miscarriages (Rai & Regan 2006), with the exclusion of identifiable causes. Inclusion criteria included negative antiphospholipid antibody profile, normal thyroid function, no evidence of hypercoagulability by thromboelastography and no anatomical malformations on either saline infusion sonography or hysteroscopy. Repeated implantation failure (RIF) was defined as two or more failed transfers of good-quality embryos (grade A/B blastocysts) in the absence of any identifiable cause (Simon & Laufer 2012). Pre-implantation genetic testing for aneuploidy (PGT-A) was not used for any case during the study period. Fertile controls were recruited altruistically, from the same age range as the study cases in a 1:4 ratio, all of whom had at least one prior term delivery and no history of miscarriage or infertility. Fifty-eight patients with defined RPL/RIF and 15 age-matched controls of proven fertility were immunophenotyped using menstrual blood obtained via Mooncup™ within 24 h of the start of menstruation. The ‘start’ was defined as the commencement of full menstrual flow, rather than the beginning of initial spotting. Samples were all analysed less than 24–48 h after collection, and carefully were temperature-controlled throughout, in an attempt to prevent uneven immune cell loss. 7-AAD staining confirmed minimal lymphocyte apoptosis under these conditions.

Histopaque®-1077 (SIAL, Ireland) combined with density gradient centrifugation was utilised to separate a distinct buffy coat from the rest of the material, and a fixed volume of this material (200 μL) was used in each instance, plus 200 μL of BD stain buffer for dilution and background autofluorescence suppression purposes. 200 μL of this suspended cell solution was added to each flow tube, already prepared with a standardised 100 μL of a suitable antibody cocktail as described below, incubated at room temperature for 20 min, and 900 μL Versalyse™ solution (Beckman Coulter UK Ltd.) was used to eliminate red blood cell contamination. Each antibody tube was made up to a final volume of 100 μL with BD staining buffer.

Co-localisation of selected antibodies was employed across individual tubes using flow cytometry (Navios™, Beckman Coulter UK LTD) for cellular evaluation, with an eight colour flow panel and appropriate compensation matrix as previously described (Marron *et al.* 2018*b*) (Supplementary Fig. 1, see section on supplementary materials given at the end of this article). Cell types were defined according to accepted conventions. Uterine/decidual type NK cells (uNK; CD3−, CD16−, CD56^bright^), peripheral type NKs (pNK: CD16+, CD56^dim^), T lymphocytes (CD4+ and CD8+) and B lymphocytes (CD19+) were assessed. All gates were based on side scatter plus CD45+ discrimination of lymphocytes and so excluded leukocytes (macrophages in particular) and other cellular materials present (stromal tissue, endothelial and epithelial cells, etc.). Percentage values are relative to the lymph gate such that all values will add up to 100%.

Informed consent was obtained from patients for menstrual blood procurement and immunophenotype analysis. Advanced ethical approval to commence the study was obtained from the research and quality management department. IBM SPSS v24 was employed for statistical analysis. ANOVA was used to compare the mean difference between groups.

## Results

Totally 73 subjects were recruited; 15 age-matched controls from a non-IVF population with proven fertility and 58 patients with adverse reproductive outcome, divided into either recurrent pregnancy loss (*n* = 27) or repeated implantation failure (*n* = 31). Using flow cytometric side scatter and CD45+ gating, lymphocytes could be clearly differentiated from the other cells present in the menstrual blood. Immunophenotyping revealed a diverse lymphocyte subpopulation comprising T and B cells and NK cells (Table 1). The most abundant lymphocytes were the uterine/decidual type, or uNK (CD16-, CD56^bright^), usually found in endometrial tissue rather than peripheral blood. The remaining NKs, in vastly reduced numbers, were peripheral type (pNK; CD3-, CD16+, CD56^dim^) or T-cell type (NK-T; CD3+, CD56^dim^). However, quite dramatic increases in cell percentages were observed relative to control tissue. Distinct T cell subpopulation differences are noted between menstrual and peripheral blood. T cells expressing CD4 and CD8 were seen to varying degrees in menstrual samples for all cases, but invariably the CD4:CD8 median ratio (0.48:1) was inverse to that typically seen in peripheral blood (2:1).

**Figure 1:**
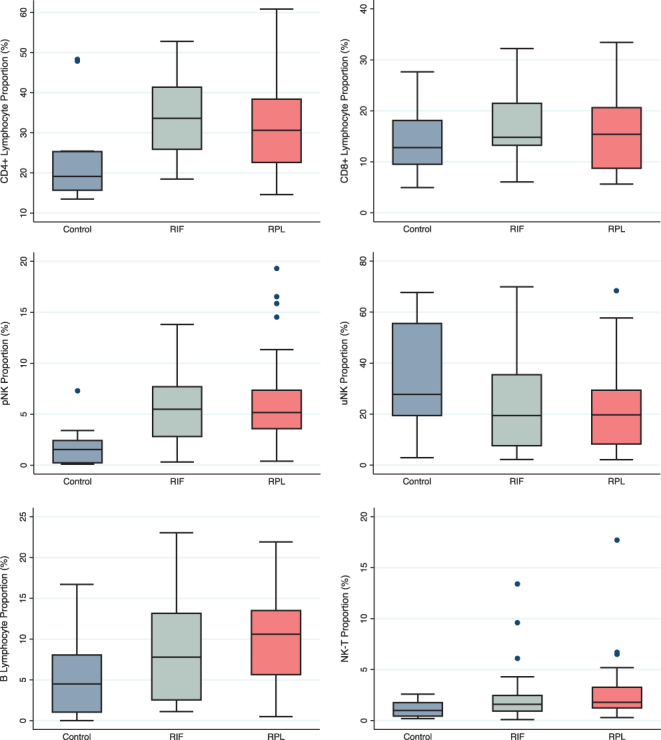
Box plot demonstrating differences in constituent lymphocyte proportions (CD4, CD8, uNK, pNK, NKT, B cell) of the menstrual blood immunophenotype (MIP) between fertile controls (Control), repeated implantation failure (RIF) and recurrent pregnancy loss (RPL) groups.

Marked differences in menstrual blood lymphocyte populations are noted between the fertile control profiles and the adverse reproductive outcome cases (Fig. 1). It is observed that significantly higher proportions of CD4+ T-Lymphocytes (*P* = 0.009), peripheral-type NK cells (*P* = 0.002) and B-lymphocytes (*P* = 0.026) are present in the RPL/RIF patient groups (Table 2). There were, however, no significant differences in the proportions of uterine NK cells (*P* = 0.163), CD8+ T-lymphocytes (*P* = 0.245) or NK-T cells (*P* = 0.189). The CD4:CD8 ratio was also higher in the RIF/RPL cases (*P* = 0.004). Reference ranges and centile charts for lymphocyte parameters are calculated based on centiles for the overall study data (Table 1). Although a significant difference was noted between the adverse reproductive outcome patients and fertile controls, any differences between recurrent pregnancy loss patients and those with repeated implantation failure did not reach statistical significance (Table 2).

**Table 1:** Population centile charts for various lymphocyte proportions in menstrual blood for determination of reference ranges.

	5th	25th	50th	75th	95th
CD4	15.1	21.2	28.9	39.3	52.3
CD8	6.3	10.8	14.7	20.4	30.3
NKT	0.3	0.7	1.7	2.5	7.9
uNK	2.8	10.8	20.3	34.2	66.0
pNK	0.2	1.7	4.3	7.4	14.9
BCell	0.5	4.2	7.8	12.8	19.9
CD4:8	1.1	1.5	1.8	2.5	3.7

**Table 2:** Mean lymphocyte proportions (with 95% CIs) in menstrual blood across the three patient populations, fertile control, repeated implantation failure (RIF) and recurrent pregnancy loss (RPL). Overall statistical significance between groups was assessed using ANOVA and subgroup comparison between RIF and RPL using two-tailed *t*-test.

Cell type	Controls	RIF	RPL	*P* values	
				ANOVA	RIF vs RPL
CD4	22.8 (16.5–28.6)	34.0 (30.3–37.8)	31.6 (27.3–36.4)	0.009*	0.42
CD8	14.1 (10.8–17.7)	17.3 (14.7–20.0	15.0 (12.6–17.4)	0.245	0.19
NKT	1.1 (0.7–1.6)	2.4 (1.3–3.6)	2.8 (1.6–4.0)	0.189	0.64
uNK	33.5 (22.1–44.7)	24.1 (16.4–31.8)	22.5 (16.5–28.4)	0.163	0.74
pNK	1.8 (0.8–2.9)	5.8 (4.3–7.3)	6.5 (4.8–8.2)	0.002*	0.62
B cell	5.3 (2.9–7.8)	9.1 (6.6–11.5)	9.9 (8.0–12.0)	0.026*	0.59
CD4:8	1.5 (1.3–1.7)	2.1 (1.9–2.4)	2.3 (2.0–2.7)	0.004*	0.44

Bold and asterisk indicate statistical significance (*P* < 0.05).

## Discussion

Menstrual blood immune cell population values in recurrent pregnancy loss and implantation failure patients have been described in detail for the first time. The cases described presented with poor reproductive history, either repeated episodes of miscarriage or unsuccessful embryo transfer, with a history of infertility. Conversely, the selected age-matched controls all had proven fertility, with no difficulty conceiving or maintaining a pregnancy. Immunophenotype differences were clearly observed in the distribution patterns of cases relative to the control population. More research is still needed to definitively establish which aspects of the immune system play a dominant role in immune-mediated implantation failure or early pregnancy loss, to support the development of more targeted therapies. RPL has a relatively low occurrence, found in only 1–4% of couples. For a subgroup of these patients, traditionally labelled as ‘unexplained’, an alloimmune aetiology mediated by dysfunctional elements within the immune system has been proposed (Rai & Regan 2006, Southcombe *et al.* 2017). Similarly, for repeated implantation failure, if more established causes have been excluded, then a potential for immunological dysfunction has also been hypothesised (Simon & Laufer 2012, Franasiak *et al.* 2021).

The complex structure of the endometrium encompasses an array of distinct molecules which contribute to cell distribution, adhesion, trafficking and signalling processes, so interactions in these areas may impact embryonic pathology (Lucas *et al.* 2016, Ticconi *et al.* 2019).

The origin of the endometrial immune cells is unclear, but they are primarily thought to be tissue resident (Franasiak *et al.* 2021); arising from progenitor CD34+ resident stem cells. Alternative hypotheses include trafficking from the peripheral blood, opening up potential new avenues for pathological investigations, such as gap junction integrity failure, which is potentially also under hormonal influence, and sex steroids are shown to play an important role in mucosal barrier integrity (Grishina *et al.* 2014, Winterhager & Kidder 2015).

Attention has focused on the innate lymphoid NK cell due to the hypothesis that it can directly target non-classical HLA-G on embryonic tissue and impact trophoblast invasion (Xu *et al.* 2020). Differing subsets of NK cells perform some of these functions, and their actions can be turned on or off depending on the cytokine milieu to which they are exposed (Long *et al.* 2013, Kumar 2018). Uterine and peripheral blood NK cells have unique differences but share some similarities. The expression of CD56 ‘dim’ and ‘bright;, presence of the lymphocyte Fc gamma type III low-affinity receptor for IgG (CD16) on pNK cells and expression of CD69 tissue residency and activation marker are some of these differences (Azargoon *et al.* 2019). Their modes of action are distinctly different under the right stimuli, but emerging evidence suggests the normally more benign tissue resident uNK can potentially mimic some of the cytotoxic pNK actions in certain circumstances (Yamada *et al.* 2003, Marron *et al.* 2018*b*). Cytotoxicity changes are thought to occur in events such as uterine CMV infection, but this process has not been demonstrated against a healthy embryo attempting implantation (Siewiera *et al.* 2013). The CD56^dim^ cells, particularly those which also co-express CD57, are more mature with increased cytotoxicity (Winger 2007). It has been suggested the origins of these cells may be outside the endometrium, and this may explain the cause of their pathology (Marron *et al.* 2018*b*).

Whether there is a relationship between the uNK and pNK subtypes, and thus between the blood and endometrial compartments, is highly controversial. Some data does suggest a link, following demonstration of a positive correlation of CD56 cells in blood and biopsy assessed immunohistochemically (Park *et al.* 2010, Santillán *et al.* 2015). Another NK-type cell which is also CD56^dim^ but often overlooked, perhaps because it is not easily discriminated in non-flow cytometric assays, is the CD3+ NK-T. This can co-express either CD4+, such as the pro-inflammatory type I variant (iNK-T), or the CD8+ and CD4+ type II variant. The numbers or activation levels of these various populations may also contribute to proposed causes of immune-mediated reproductive failure.

Menstrual blood immunophenotyping may have several advantages over reproductive immunophenotyping using peripheral blood or endometrial biopsy. First, it is non-invasive and can therefore be in a conceptional cycle, avoiding both risk and treatment delay in time-sensitive ART cycles. Comparative studies also indicate that menstrual blood-derived organoids match those obtained using endometrial biopsy (Cindrova-Davies *et al.* 2021). This method can also be easily expanded to simultaneously assess other factors that may interfere with the implantation of viable embryos, such as viral components (e.g. HHV6a), past STI’s, microbiome/endometritis evaluations, endometriosis and endometrial receptivity. Compared to peripheral blood, the dominance of CD8+ T cells over CD4+ is described as similar to luteal phase endometrial tissue, indicating low levels of peripheral blood contamination in our method (Southcombe *et al.* 2017, Marron *et al.* 2018*b*, Marron & Harrity 2019). Increases in CD4 expression in the endometrial biopsy itself are associated with a negative outcome and are often seen in RIF and particularly RPL. In this regard, raised CD4+ levels may be associated with RPL, while low CD8 may be a prognostic marker for RIF (El-Shahawy 2016, Marron & Harrity 2019).

Immunotherapy for implantation failure or miscarriage is perceived as a controversial strategy, with concerns regarding the lack of strong evidence to support routine use (Hviid & Macklon 2017). A common theme to many of these reservations is poor study design and patient selection, which perhaps devalues data showing a significant impact of therapy on outcomes such as live birth (Alecsandru & Garcia-Velasco 2015) or adds fears of bias during patient selection. A targeted and validated non-invasive assessment, such as menstrual immunophenotyping, could be utilised to better identify patient subgroups with poor reproductive history who are more likely to benefit from a greater degree from targeted immunotherapy (Sacks 2015). Further studies are required on these specific patients to assess if specific immune treatments, personalised to the local environment, could have a role in the successful treatment of these individuals.

## Supplementary Material

Supplementary Figure. Fluorophores used for Flow Cytometry (Navios™ Flow cytometer, Beckman Coulter, UK LTD)

## Declaration of interest

The authors declare that there is no conflict of interest that could be perceived as prejudicing the impartiality of the research reported.

## Funding

This work did not receive any specific grant from any funding agency in the public, commercial, or not-for-profit sector.

## Author contribution statement

K M conceived and performed the study and wrote the paper. C H performed statistics and edited the manuscript.
