# Supplementary material for: Potential utility of a non-invasive menstrual blood immunophenotype analysis in reproductive medicine
Source: Reprod Fertil. 2022 Sep 16;3(4):255–61. doi: 10.1530/RAF-22-0047 (PMC9641796; doi:10.1530/RAF-22-0047)
Supplement: Supplementary Figure. Fluorophores used for Flow Cytometry (Navios™ Flow cytometer, Beckman Coulter, UK LTD) [file supplementary_figure_1.pdf]

Laser	405 Excitation		488 Excitation				633 Excitation	
Flurophore	Pacific Blue	Krome Orange	PE	ECD	PC7	PC5.5	APC-AF700	APC-AF750
CD marker	**CD3**	**CD45**	**CD56**	**CD16**	**CD8**	**CD19**	**CD5**	**CD4**
Clone	UCHT1	J.33	N901	3G8	SFC121	J3-119	BL1a	13B8.2

Supplementary Figure.

Fluorophores used for Flow Cytometry (Navios™ Flow cytometer, Beckman Coulter, UK LTD)
